# Unveiling anti-oxidative and anti-inflammatory effects of docosahexaenoic acid and its lipid peroxidation product on lipopolysaccharide-stimulated BV-2 microglial cells

**DOI:** 10.1186/s12974-018-1232-3

**Published:** 2018-07-09

**Authors:** Bo Yang, Runting Li, C. Michael Greenlief, Kevin L. Fritsche, Zezong Gu, Jiankun Cui, James C. Lee, David Q. Beversdorf, Grace Y. Sun

**Affiliations:** 1 Chemistry Department, University of Missouri, Columbia, MO USA; 2 Biochemistry Department, University of Missouri, 117 Schweitzer Hall, Columbia, MO 65211 USA; 3 Department of Nutrition and Exercise Physiology, University of Missouri, Columbia, MO USA; 4 Department of Pathology and Anatomical Sciences, University of Missouri, Columbia, MO USA; 5 Department of Bioengineering, University of Illinois at Chicago, Chicago, IL USA; 6 Departments of Radiology, Neurology and Psychological Sciences, University of Missouri, Columbia, MO USA

**Keywords:** Docosahexaenoic acid (DHA), 4-Hydroxyhexenal (4-HHE), 4-Hydroxynonenal (4-HNE), Microglia, Lipopolysaccharide (LPS), NO, ROS, Nrf2, HO-1, cPLA_2_, iPLA_2_

## Abstract

**Background:**

Phospholipids in the central nervous system are enriched in n-3 and n-6 polyunsaturated fatty acids (PUFA), especially docosahexaenoic acid (DHA) and arachidonic acid (ARA). These PUFA can undergo enzymatic reactions to produce lipid mediators, as well as reaction with oxygen free radicals to produce 4-hydroxyhexenal (4-HHE) from DHA and 4-hydroxynonenal (4-HNE) from ARA. Recent studies demonstrated pleiotropic properties of these peroxidation products through interaction with oxidative and anti-oxidant response pathways. In this study, BV-2 microglial cells were used to investigate ability for DHA, 4-HHE, and 4-HNE to stimulate the anti-oxidant stress responses involving the nuclear factor erythroid-2-related factor 2 (Nrf2) pathway and synthesis of heme oxygenase (HO-1), as well as to mitigate lipopolysaccharide (LPS)-induced nitric oxide (NO), reactive oxygen species (ROS), and cytosolic phospholipase A_2_ (cPLA_2_). In addition, LC-MS/MS analysis was carried out to examine effects of exogenous DHA and LPS stimulation on endogenous 4-HHE and 4-HNE levels in BV-2 microglial cells.

**Methods:**

Effects of DHA, 4-HHE, and 4-HNE on LPS-induced NO production was determined using the Griess reagent. LPS-induced ROS production was measured using CM-H_2_DCFDA. Western blots were used to analyze expression of p-cPLA_2_, Nrf2, and HO-1. Cell viability and cytotoxicity were measured using the WST-1 assay, and cell protein concentrations were measured using the BCA protein assay kit. An ultra-high-performance liquid chromatography-tandem mass spectrometry (LC-MS/MS) analysis was used to determine levels of free 4-HHE and 4-HNE in cells.

**Results:**

DHA (12.5–100 μM), 4-HHE (1.25–10 μM), and 4-HNE (1.25–10 μM) dose dependently suppressed LPS-induced production of NO, ROS, and as p-cPLA_2_ in BV-2 microglial cells. With the same concentrations, these compounds could enhance Nrf2 and HO-1 expression in these cells. Based on the estimated IC_50_ values, 4-HHE and 4-HNE were five- to tenfold more potent than DHA in inhibiting LPS-induced NO, ROS, and p-cPLA_2_. LC-MS/MS analysis indicated ability for DHA (10–50 μM) to increase levels of 4-HHE and attenuate levels of 4-HNE in BV-2 microglial cells. Stimulation of cells with LPS caused an increase in 4-HNE which could be abrogated by cPLA_2_ inhibitor. In contrast, bromoenol lactone (BEL), a specific inhibitor for the Ca^2+^-independent phospholipase A_2_ (iPLA_2_), could only partially suppress levels of 4-HHE induced by DHA or DHA + LPS.

**Conclusions:**

This study demonstrated the ability of DHA and its lipid peroxidation products, namely, 4-HHE and 4-HNE at 1.25–10 μM, to enhance Nrf2/HO-1 and mitigate LPS-induced NO, ROS, and p-cPLA_2_ in BV-2 microglial cells. In addition, LC-MS/MS analysis of the levels of 4-HHE and 4-HNE in microglial cells demonstrates that increases in production of 4-HHE from DHA and 4-HNE from LPS are mediated by different mechanisms.

## Background

The high abundance of n-3 polyunsaturated fatty acids (PUFA) in the phospholipids in the brain and retina has led to immense interest in the search of their functional roles in health and diseases in these organs [1]. A number of studies with animal models have demonstrated the ability of docosahexaenoic acid (DHA) to protect the brain against aging and neurodegenerative diseases, including stroke, spinal cord injury, and traumatic brain injury [2–6]. Studies with human populations have also demonstrated effects of n-3 PUFA to improve neurologic functions such as depression, cognition, and memory [7]. To date, n-3 PUFA in the form of fish oil are probably one of the most highly consumed supplements by humans. Nevertheless, despite of a possible link between the n-3 PUFA and neural functions, the underlying mechanisms for the beneficial effects remain elusive [8].

Based on the “deacylation-reacylation” hypothesis, the PUFA in membrane phospholipids, in particular, the arachidonic acid (ARA) and DHA, may become metabolically active upon release by phospholipases A_2_ (PLA_2_) [9]. Studies have demonstrated the release of ARA through the action of cPLA_2_, a Ca^2+^-dependent enzyme with active phosphorylation sites linked to the mitogen-activated protein kinases (MAPKs) [9]. In microglial cells, activation of cPLA_2_ has been linked to inflammatory responses, increases in reactive oxygen species (ROS), and inducible nitric oxide synthase (iNOS) [10]. In contrast, the release of DHA is mediated by the action of Ca^2+^-independent PLA_2_ (iPLA_2_) [11–14]. While ARA is metabolized by cyclooxygenases (COX) and lipoxygenases (LOX) to produce eicosanoids, which are inflammatory mediators, DHA is metabolized by specific LOX to form oxylipins which are generally protective [1].

Besides the enzymatic pathways, these PUFA are also substrates of non-enzymatic action through peroxidation by radiation and ROS generated from extracellular and intracellular sources. Lipid peroxidation products such as 4-hydroxynonenal (4-HNE) from ARA and 4-hydroxyhexenal (4-HHE) from DHA are bioactive molecules capable of forming adducts with DNA, proteins, and lipids [15–17]. Increases in lipid peroxidation have been implicated in neuroinflammatory diseases [18, 19]. In fact, 4-HNE protein adducts have been used as a marker for different types of brain injury [16]. Although less is known about adducts with 4-HHE, advances in studies using LC-MS/MS methods have identified these 4-hydroxy alkenals in tissues, plasma, and urine [20–24]. Due to their electrophilic properties [25], there is evidence that these 4-hydroxy alkenals can engage in adaptive responses by upregulating the anti-oxidant response pathway involving Nrf2 which leads to increases in synthesis of phase II enzymes, including HO-1 [20, 24, 26–28].

Microglia are important immune cells in the brain, and their activation has been implicated in a number of neurodegenerative diseases and different types of brain injury. In recent years, numerous studies have used murine immortalized BV-2 microglial cells as a model to investigate inflammatory and oxidative responses. Our previous studies showed that BV-2 cells exhibit many properties similar to primary microglial cells isolated from 7- to 10-day-old C57BL/6 pup brains using the magnetic activated cell sorting protocol [29]. In this study, primary microglial cells were isolated using a cluster of differentiation molecule CD11b microbeads. Cells were cultured in DMEM supplemented with 10% FBS containing 100 units/mL penicillin and streptomycin, and culture medium was replaced every 3–5 days and cultures were used between 5 and 7 days. Our results showed that both primary microglia and BV-2 cells exhibited active response to LPS and interferon gamma (IFNγ) which induced time-dependent increases in ERK-1/2, p-cPLA_2_, and iNOS [29]. However, despite the similarities in these responses, subtle differences could be found in the extent and time of the responses (see Fig. 2 in Chuang’s paper). Based on the presence of Toll-like receptors as well as mitogen-activated protein kinase (MAPK)-induced cPLA_2_ pathways in both primary and BV-2 cells, numerous studies have used BV-2 cells to study the effects of botanical polyphenols and other factors on neuroinflammation [29–31].

Despite an yet unknown mechanism, a number of studies demonstrated the ability for DHA and its oxidative products to modulate the oxidant and inflammatory responses in microglial cells [32–36]. Studies on the vascular cell system also demonstrated ability for DHA to inhibit inflammation by modulating the cross-talk between the Nrf2/HO-1 and the NF-κB pathways [37]. In endothelial cells, relatively low concentrations of 4-HHE added exogenously could upregulate the Nrf2 pathway and increase synthesis of HO-1, a potent anti-oxidative enzyme [38, 39]. However, a systemic comparison of the effects of DHA, 4-HHE, and 4-HNE on the Nrf2/HO-1 and LPS-induced inflammatory responses in BV-2 microglial cells has not been investigated. In addition, little is known whether the endogenous lipid peroxidation products in these cells can be regulated by exogenous DHA and stimulation with LPS. Here, BV-2 cells were used to examine the effects of exogenous DHA, 4-HHE, and 4-HNE to enhance the Nrf2 and HO-1 pathway, and to mitigate LPS-induced NO, ROS, and cPLA_2_. Subsequently, using LC-MS/MS analysis to measure intracellular levels of 4-HHE and 4-HNE in the BV-2 cells, studies were performed to evaluate mechanisms for DHA and LPS on these peroxidation products.

## Methods

### Materials

Dulbecco’s modified Eagle’s medium (DMEM) and penicillin/streptomycin were obtained from GIBCO (Gaithersburg, MD). U0126 inhibitor was purchased from Cell Signaling (Beverly, MA). Arachidonyl trifluoromethyl ketone (ATK, AACOCF3), racemic bromoenol lactone (BEL), 4-hydroxyhexenal (4-HHE, 1 mg in 0.1 mL of ethanol), 4-hydroxynonenal (4-HNE, 1 mg in 0.1 mL of ethanol), 4-hydroxyhexenal-d_3_ (4-HHE-d_3_, 100 μg in 0.1 mL of methyl acetate), and docosahexaenoic acid (DHA, 100 mg in 0.4 mL of ethanol) were purchased from Cayman Chemical (Ann Arbor, MI). Fetal bovine albumin (FBS), Greiss reagent (sulfanilamide and *N*-1-napthylethylenediamine dihydrochloride), 1, 3-cyclohexanedione (CHD, 97%), ammonium acetate (HPLC grade), acetic acid (ACS grade), and formic acid (mass spectrometry grade) were purchased from Sigma-Aldrich (St. Louis, MO). For ROS detection, CM-H_2_DCFDA was purchased from Invitrogen Inc. (Eugene, OR). WST-1 assay was purchased from Clontech (Mountain View, CA). Antibodies used include the following: Nrf2 and HO-1 from Santa Cruz Biotechnology (Santa Cruz, CA), p-cPLA_2_ and cPLA_2_ from Cell Signaling (Beverly, MA), and monoclonal anti-β-actin peroxidase from Sigma-Aldrich (St. Louis, MO). C18 Sep-Pak cartridges (1 mL, 100 mg) were obtained from Waters Corporation (Milford, MA). Phospholipid removal cartridges (Phree™, 1 mL) were purchased from Phenomenex Inc. (Torrance, CA). All solvents (HPLC grade) used for LC and MS analysis were obtained from Thermo Fisher Scientific Inc. (Fair Lawn, NJ).

### Cell culture and treatments

BV-2 microglial cells were used between 14 and 25 passages and were prepared as previously described [40]. Initially, cells were cultured in 75-cm^2^ flasks with DMEM supplemented with 5% FBS containing 100 units/mL penicillin and streptomycin (100 μg/mL), and maintained in a 5% CO_2_ incubator at 37 °C. For experiments involving assays of NO and ROS, cells were subcultured in 96-well plates; for Western blot analysis, cells were subcultured in 12-well plates, and for LC-MS/MS analysis, cells were subcultured in 60-mm dishes. In all conditions, cells were subcultured to 80–90% confluence and then serum starved for 3 h prior to adding DHA and inhibitors for 1 h, and followed by stimulation with LPS (100 ng/mL). DHA was suspended in DMEM with 2% fatty acid-free BSA (Sigma-Aldrich, St. Louis, MO), and different concentrations were added to cells. Controls received DMEM with BSA without DHA. 4-HHE and 4-HNE were dissolved in dimethyl sulfoxide (DMSO). The following LPS treatment time schedule was used for different assays: 16 h for NO production, 12 h for assay of ROS, 4 h for assay of p-cPLA_2_/cPLA_2_, and 6 h for assay of Nrf2 and HO-1. For LC-MS/MS analysis of 4-HHE and 4-HNE, cells were incubated for 6 h.

### Assessment of cell viability

Cell viability was determined using the WST-1 protocol (Clontech Laboratories, Inc., Mountain View, CA). Cells were treated with DHA or alkenals for 17 h with or without LPS. WST-1 was added into each well (96-well plates) with medium/WST-1 reagent at 15:1 (*v*/*v*). Cells were incubated for 1 h at 37 °C, and after dissolving the formazan dye with DMSO, absorption was read at 450 nm using a Synergy4 Plate Reader (BioTek Instruments, Inc., St. Louis, MO).

### Measurement of ROS production

ROS production was measured using the ROS detection reagent CM-H_2_DCFDA (DCF) (Invitrogen Inc., Eugene, OR) as described previously [41]. Briefly, BV-2 cells were seeded in a 96-well plates for 24 h. Cells were then starved for 3 h in the serum-free DMEM prior to adding DHA (1 h) and followed by addition of LPS for 11 h. DCF (1 μM final concentration) was added to each well for 1 h. The fluorescence intensity of DCF was measured using the Synergy4 Plate Reader with an excitation wavelength of 490 nm and an emission wavelength of 520 nm.

### NO determination in culture medium

NO released from cells was converted to nitrite in the culture medium and was determined using Griess reagent [42]. Cells were cultured in phenol red free DMEM. After treatment with LPS for 16 h, aliquots (50 μL) of culture medium were transferred to 96-well plates and incubated with 50 μL of reagent A [1% (*w*/*v*) sulfanilamide (Sigma-Aldrich, St. Louis, MO) in 5% phosphoric acid] for 10 min at room temperature in the dark, and followed by incubation with 50 μL of reagent B [0.1%, *w*/*v*, *N*-1-napthylethylenediamine dihydrochloride (Sigma-Aldrich, St. Louis, MO)] for 10 min at room temperature in the dark. The absorbance of samples was measured at a wavelength of 565 nm using the Synergy4 Plate Reader. Sodium nitrite (0–100 μM), diluted in culture media, was used to prepare the nitrite standard curve.

### Western blot analyses

Western blot analysis was carried out as described earlier [29]. Briefly, after various treatment protocols, cells were harvested in Laemmli lysis buffer and centrifuged at 10,000 × *g* for 15 min at 4 °C to remove cell debris. After protein quantification with the BCA protein assay kit (Pierce Biotechnology, Rockford, IL), samples together with Precision Plus Protein standards (Dual color, BioRad, Hercules, CA) were loaded in sodium dodecyl sulfate-polyacrylamide gel electrophoresis (SDS-PAGE) gels and resolved at 100 V. After electrophoresis, proteins were transferred to 0.45-μm nitrocellulose membranes at 100 V for 1.5 h. Membrane strips were blocked in Tris-buffered saline (TBS), pH 7.4, with 0.1% Tween 20 (TBS-T) containing 5% non-fat milk for 1.5 h at room temperature. The blots were incubated with anti-Nrf2 (1:500 dilution), anti-HO-1 (1:800 dilution), or p-cPLA_2_ (1:1000 dilution) or cPLA_2_ (1:1000 dilution) antibodies overnight at 4 °C. After repeated washing with TBS-T, blots were incubated with goat anti-rabbit IgG-horseradish peroxidase (1:6000 dilution) for 1 h at room temperature and then washed three times with TBS-T. Immuno-labeling was detected by SuperSignal chemiluminescent substrates (Thermo Scientific, Rockford, IL). For loading control, blots were incubated with anti-β-actin (1:50,000) and goat anti-mouse IgG-horseradish peroxidase (1:6000). Films were scanned, and the optical density of protein bands was measured using the QuantityOne software program (BioRad, Hercules, CA).

### Quantitative analysis of 4-HHE and 4-HNE in microglial cells

Cells were subcultured in 60-mm dishes, and after different treatment conditions, the medium was removed and 0.5 mL of phosphate-buffered saline (PBS)-methanol (1:1, *v*/*v*) was added. Cells were harvested by scraping and then transferred into an Eppendorf tube. The cell suspension was mixed, and aliquots were taken for protein determination and for determination of 4-HHE and 4-HNE using LC-MS/MS. For the analysis, 30 μL of analyte was added to equal volume of internal standard (4-HHE-d_3_, 1000 ng/mL), and acetonitrile (0.5 mL) containing 1% formic acid was added to the mixture to precipitate the proteins. Solid-phase extraction (SPE) was carried out using a Phree™ cartridge to remove the phospholipids [43]. After evaporation by nitrogen to remove the excess solvent, samples were dissolved in 20 μL of methanol, followed by adding 200 μL of freshly prepared acidified 1,3-cyclohexanedione (CHD) reagent [44]. In brief, CHD (25 mg), ammonium acetate (1 g), and acetic acid (0.5 mL) were dissolved in HPLC water to a total volume of 10 mL. The pale yellow solution (pH 5.0) was incubated at 60 °C for 1 h and cooled on ice prior to addition. The derivatization reaction mixture was heated at 60 °C for another 1 h, cooled on ice, and then desalted through a C18 SPE cartridge. The C18 cartridge was pre-conditioned with methanol (0.7 mL) and water (0.7 mL). The reaction mixture (of 220 μL total volume) was loaded onto the cartridge, followed by washing twice with water (0.7 mL) and finalized with 5% acetonitrile in water (0.7 mL). 4-HHE and 4-HNE derivatives were eluted with 100% acetonitrile (0.7 mL) and dried with a steady stream of nitrogen gas. The samples were reconstituted in 40% methanol in water (300 μL) containing 0.1% formic acid and were ready for LC-MS/MS analysis.

A Waters Acquity LC system equipped with a quaternary solvent manager was used in conjunction with a C18 column (Acquity CSH, 1.7 μm, 100 × 2.1 mm^2^, Waters, Milford, MA, USA). A LC method was developed and optimized to separate the 4-HHE and 4-HNE derivatives with a 7-min LC run time including equilibration. Water containing 0.1% formic acid (solution A) and methanol containing 0.1% formic acid (solution B) were used as the mobile phases. The solvent gradient was 60% A 40% B at 0 min, 60% A 40% B at 0.25 min, 10% A 90% B at 3.55 min, 10% A 90% B at 3.80 min, 60% A 40% B at 3.90 min, and 60% A 40% B at 7.00 min. The flow rate was 0.3 mL/min, and the injection volume was 10 μL in the full-loop mode. The column was heated to 40 °C, and the sample chamber was maintained at 10 °C.

A Waters Xevo TQ-S triple quadrupole mass spectrometer with electrospray ionization, operated in the positive-ionization mode, was used. The multiple reaction monitoring (MRM) scans were conducted by selecting parent and daughter ion pairs of a specific analyte, which were optimized using the IntelliStart™ software. The 4-HNE derivatives exhibited an abundant pseudo parent ion, [M-18+H]^+^ at m/z 326.3 Da. The dehydrated parent ion generates a major principal daughter ion at m/z 216.1 Da. The MRM transition m/z 326.3 to > 216.1 Da showed higher sensitivity than other transitions and consequently was chosen as the quantifying transition for the 4-HNE derivative. Similarly, the MRM transitions m/z 284.2 to > 216.1 Da and 287.2 to > 216.1 Da were chosen for simultaneous monitoring of 4-HHE and 4-HHE-d_3_ (internal standard) derivatives, respectively. The desolvation temperature was 350 °C, and the source temperature was 150 °C. A capillary voltage of 3.8 kV, cone voltage of 40 V, and collision energy of 25 eV were used. The nebulizer gas rate was 800 L N_2_/h. MassLynx software (Version 4.1, Waters) was used for all data acquisition.

Because of the presence of endogenous 4-HHE and 4-HNE in the cell suspension, aqueous solutions were used to prepare the calibration standards. All the stock solutions (50 μg/mL) were prepared in methanol. A working solution containing 2.5 μg/mL of 4-HHE was prepared by diluting the stock solution with methanol. The internal standard working solution (1000 ng/mL) was prepared in methanol. Calibration curves were prepared by spiking the 4-HHE working solution into aqueous solution and preparing serial dilutions that yielded seven calibration standards (15.63, 31.25, 62.50, 125.0, 250.0, 500.0, and 1000 ng/mL). Similar calibration standards with the same spiking concentrations but in cell suspensions for 4-HHE were also prepared and used to assess any difference between standard curves prepared using aqueous solution or cell suspensions. The results showed the same response for 4-HHE in the two solvent systems and that calibration curves prepared in aqueous solution are suitable for analysis of the biological samples. Similarly, seven calibration standards (6.25, 12.5, 25.0, 50.0, 100, 125, and 250 ng/mL) of 4-HNE were prepared as described, and no difference between solvent systems was observed.

A full method for validation was performed, including analysis of limit of quantification, linearity, recovery, matrix effect, and intraday/interday reproducibility as previously described [45]. Briefly, the lower limit of quantification (LLOQ) is defined as a signal-to-noise ratio of 10:1 for a given MRM chromatogram. Recovery of phospholipid removal by the SPE cartridge was determined by comparing the ratio of peak areas of analyte to the internal standard peak area for samples spiked before SPE to the ratio of peak areas of analyte to internal standard spiked after SPE. Recovery of the C18 SPE cartridge was accessed by comparing the ratio of peak areas of analyte to the internal standard for samples prior to SPE to the ratio of peak areas of analyte to internal standard spiked after SPE. Because of the presence of endogenous levels of 4-HHE and 4-HNE in the cell suspension, the potential matrix effects of the cell suspension were analyzed by comparing the peak area of the spiked 4-HHE-d_3_ in cell suspension to neat standard prepared at the same concentration in methanol. The precision and accuracy of interday and intraday reproducibility were analyzed by performing analysis on three different batches of quality control samples over 3 days.

### Statistical analysis

For studies to assess oxidative and inflammatory responses, triplicate analyses were performed on a given sample and at least three independent experiments (with different passages) were performed for each condition. For studies measuring 4-HHE and 4-HNE levels using the LC-MS/MS analysis, cells in each passage were cultured in 60-mm dishes (in triplicate) and assays of alkenals from each extract were carried out in duplicate. Results are expressed as standard error of the mean (SEM) and analyzed by one-way ANOVA followed by Bonferroni post-tests (v7.00; GraphPad Prism Software Inc., San Diego, CA). When two groups were compared, differences were analyzed by a two-tailed Student’s *t* test. Differences were considered significant at *p* < 0.05 for all analyses.

## Results

### Effects of DHA, 4-HHE, and 4-HNE on BV-2 cell viability

**Fig. 1:**
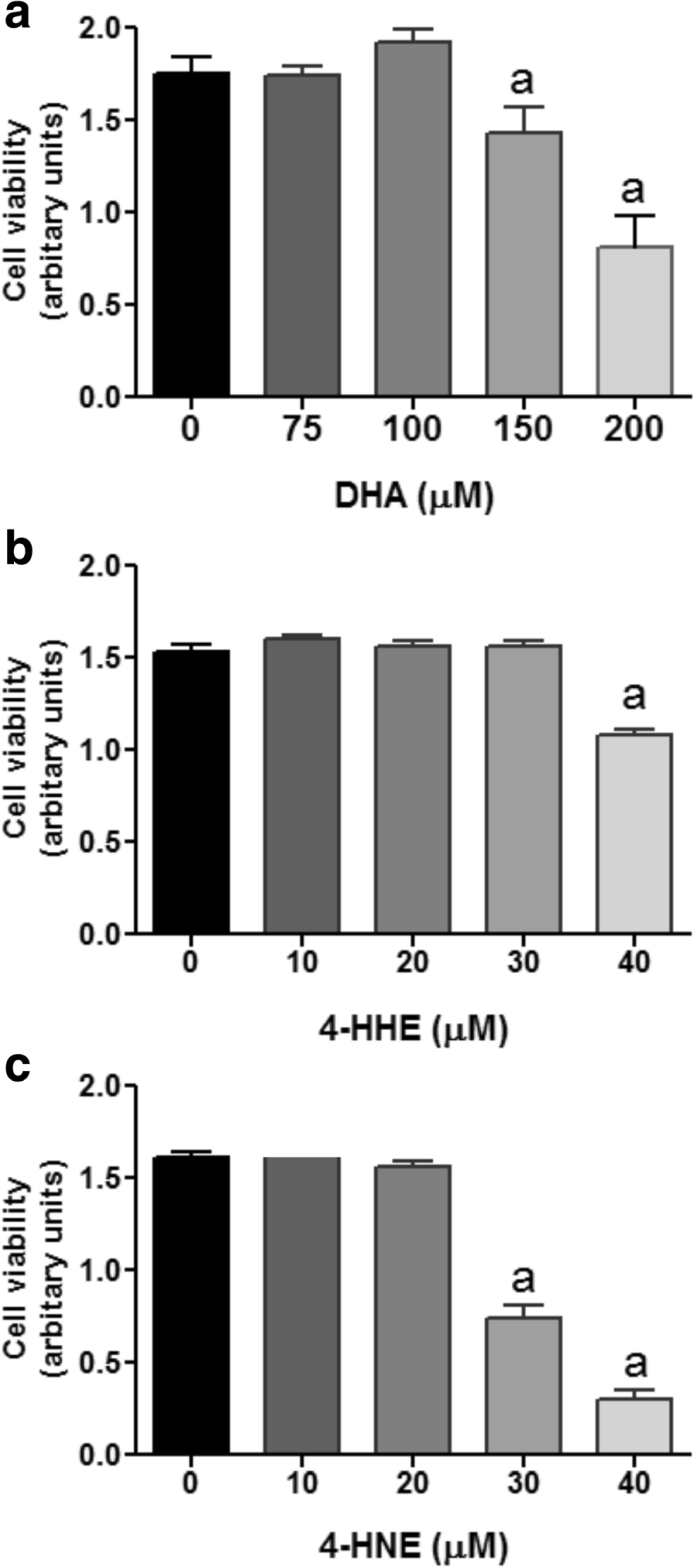
**a**–**c** Effects of DHA, 4-HHE, and 4-HNE on viability of BV-2 microglial cells. BV-2 microglial cells were cultured in 96-well plates until reaching 90% confluence. Cells were serum-starved for 3 h, and DHA, 4-HHE, and 4-HNE at different concentrations were added for 1 h. After incubation for 16 h, cells were taken for viability assay by incubating with the WST-1 reagent at 37 °C for 30 min. Color density was read at 450 nm with the plate reader. Results were obtained from triplicate assay from a single passage and expressed as mean ± SE (*n* = 3). Repeated experiment with different passages showed similar results. Analysis by one-way ANOVA followed by Bonferroni post-tests; “a” represents significant differences (*p* < 0.05) comparing test compounds with control

In this study, the WST-1 assay was used to test the concentrations of DHA, 4-HHE, and 4-HNE to confer cytotoxic effects in BV-2 microglial cells. Cells were serum starved for 3 h and followed by incubation of test compounds at different concentrations for 16 h. This time point was also used for the NO assay. As shown in Fig. 1a, cytotoxic effects for DHA were observed with concentrations greater than 150 μM. Figure 1b, c shows cell viability decreasing for 4-HHE at concentrations higher than 40 μM and for 4-HNE at concentrations higher than 30 μM. Based on these results, subsequent studies to test the effects of these compounds were carried out below the cytotoxic concentrations.

### DHA, 4-HHE, and 4-HNE suppress LPS-induced NO production in microglial cells

**Fig. 2:**
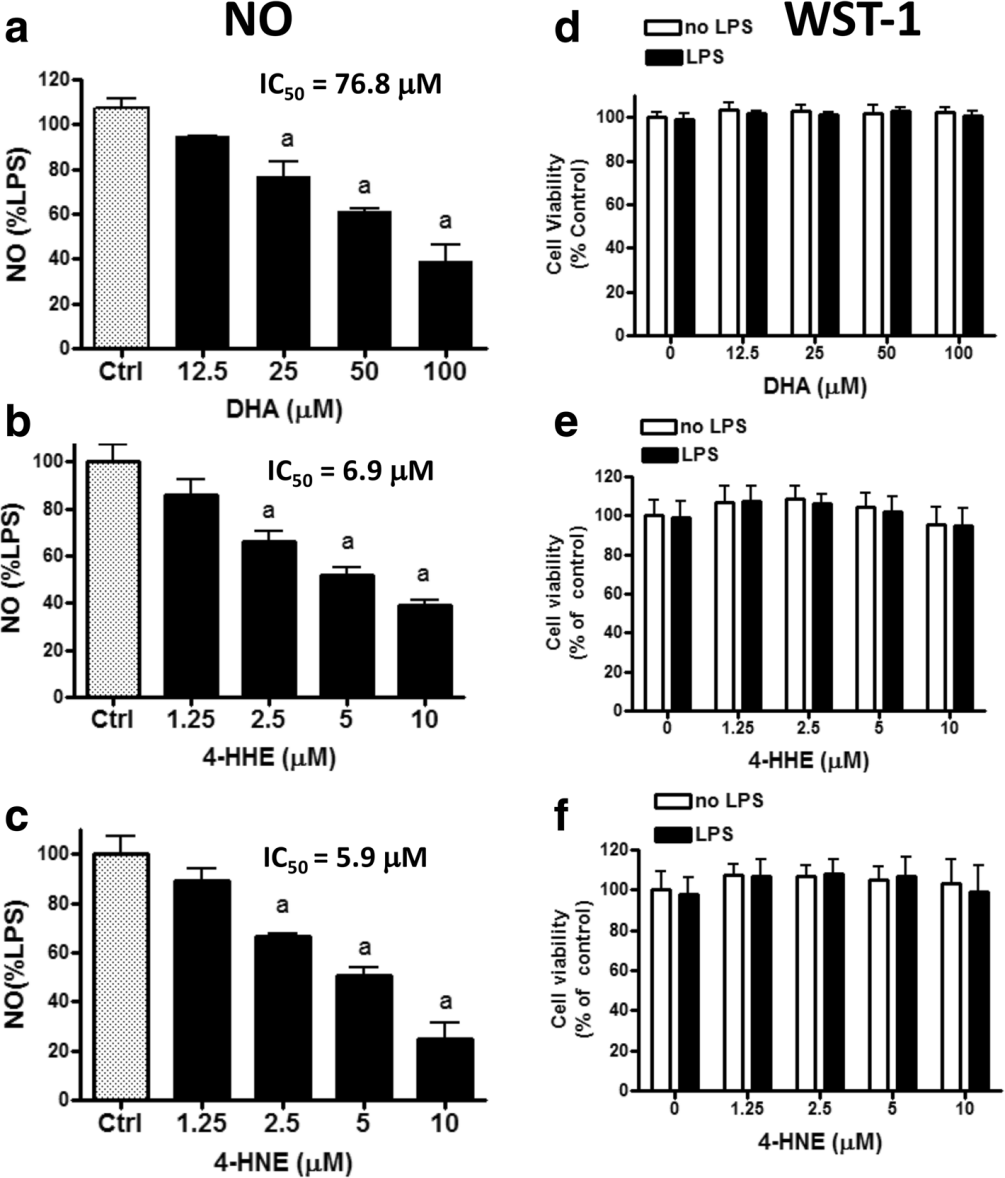
Effects of DHA, 4-HHE, and 4-HNE on LPS-induced NO production in BV-2 microglial cells. BV-2 microglial cells (10^6^) were subculture in 96-well plates to 80% confluent. At the time of experiment, cells were serum-starved for 3 h and pre-treated with DHA (12.5–100 μM), 4-HHE (1.25–10 μM), and 4-HNE (1.25–10 μM) for 1 h, and followed by stimulation with LPS (100 ng/mL) for 16 h. Data in **a**–**c** represent inhibition of LPS-induced NO production by DHA, 4-HHE, and 4-HNE using the Griess reagent. Results were obtained from triplicate assay from each cell passage. Concentrations of NO in μM ± SE (*n* = 5) were as follows: DHA 9.75 ± 1.8, 4-HHE 9.30 ± 0.10, and 4-HNE 10.43 ± 0. 52. Data in **d**–**f** represent cell viability after 16-h incubation using the WST-1 assay. Results are expressed as the mean ± SEM (*n* = 3–5) and analyzed by one-way ANOVA followed by Bonferroni post-tests; “a” represents significant differences (*p* < 0.05) comparing test compounds with control (Ctrl) with LPS treatment alone. IC_50_ values for each test compound were determined using the formula for regression analysis in Microsoft Excel 2016

In this study, we tested the effects of DHA as well as 4-HHE and 4-HNE on LPS-induced NO production in BV-2 cells. Figure 2a shows that DHA (12.5–100 μM) diminished LPS-induced NO production in a dose-dependent manner with significant decrease (*p* < 0.05) noted at concentrations > 25 μM and an IC_50_ of 76.8 μM. Under this condition, incubation of cells with DHA at different concentrations followed by LPS treatment did not alter cell viability as demonstrated by WST-1 assay (Fig. 2d).

Initial studies to test the effects of 4-HHE and 4-HNE on LPS-mediated oxidative and inflammatory responses in microglial cells indicated an effective dose range of 1.25 to 10 μM. As shown in Fig. 2b, c, 4-HHE (1.25–10 μM) and 4-HNE (1.25–10 μM) reduced LPS-induced NO production in a dose-dependent manner with significant reduction (*p* < 0.05) at concentrations more than 2.5 μM. The IC_50_ value for 4-HHE to reduce LPS-induced NO was 6.9 μM and that for 4-HNE was 5.9 μM. At these conditions, neither 4-HHE nor 4-HNE showed cytotoxic effects to the cells (Fig. 2e, f).

### DHA, 4-HHE, and 4-HNE suppress LPS-induced production of ROS

**Fig. 3:**
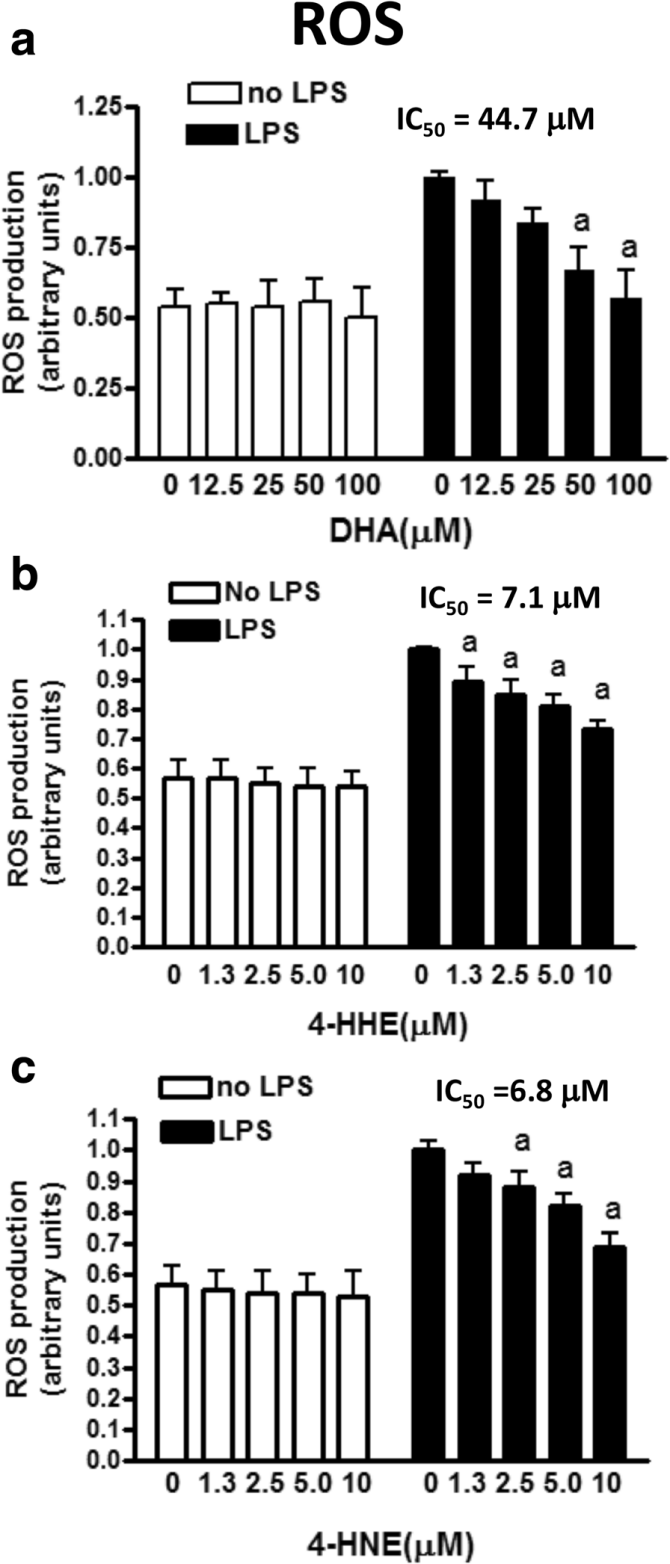
Effects of DHA, 4-HHE, and 4-HNE on LPS-induced ROS production in microglial cells. Cells were cultured in 96-well plates as described in Fig. 2 and harvested at 12 h after LPS treatment. For ROS determination, CM-H_2_DCFDA was added 1 h prior to the end of incubation. Data in **a**–**c** represent levels of ROS after pre-treatment of DHA (12.5–100 μM), 4-HHE (1.25–10 μM), and 4-HNE (1.25–10 μM) in the presence and absence of LPS. Results are expressed as the mean ± SEM (*n* = 4). Analyzed by one-way ANOVA followed by Bonferroni post-tests; “a” represents significant differences (*p* < 0.05) comparing test compounds with control (Ctrl) with LPS treatment alone. IC_50_ values for each test compound were determined using the formula for regression analysis in Microsoft Excel 2016

Using CM-H_2_DCFDA, we examined the effects of DHA (12.5–100 μM), 4-HHE (1.25–10 μM), and 4-HNE (1.25–10 μM) on ROS production in microglial cells incubated for 12 h with or without LPS (100 ng/mL). As shown in Fig. 3a, DHA suppressed LPS-induced ROS in a dose-dependent manner with significant decrease (*p* < 0.05) at 50 μM or higher. Estimation of IC_50_ for DHA indicated a value of 44.7 μM (Fig. 3a). Treatment with 4-HHE (1.25–10 μM) and 4-HNE (1.25–10 μM) also reduced LPS-induced ROS with IC_50_ of 7.1 and 6.8 μM, respectively (Fig. 3b, c). Treatment with DHA, 4-HHE, or 4-HNE alone without LPS did not alter endogenous ROS levels in the cells (Fig. 3a–c).

### DHA, 4-HHE, and 4-HNE suppress LPS-induced p-cPLA_2_

**Fig. 4:**
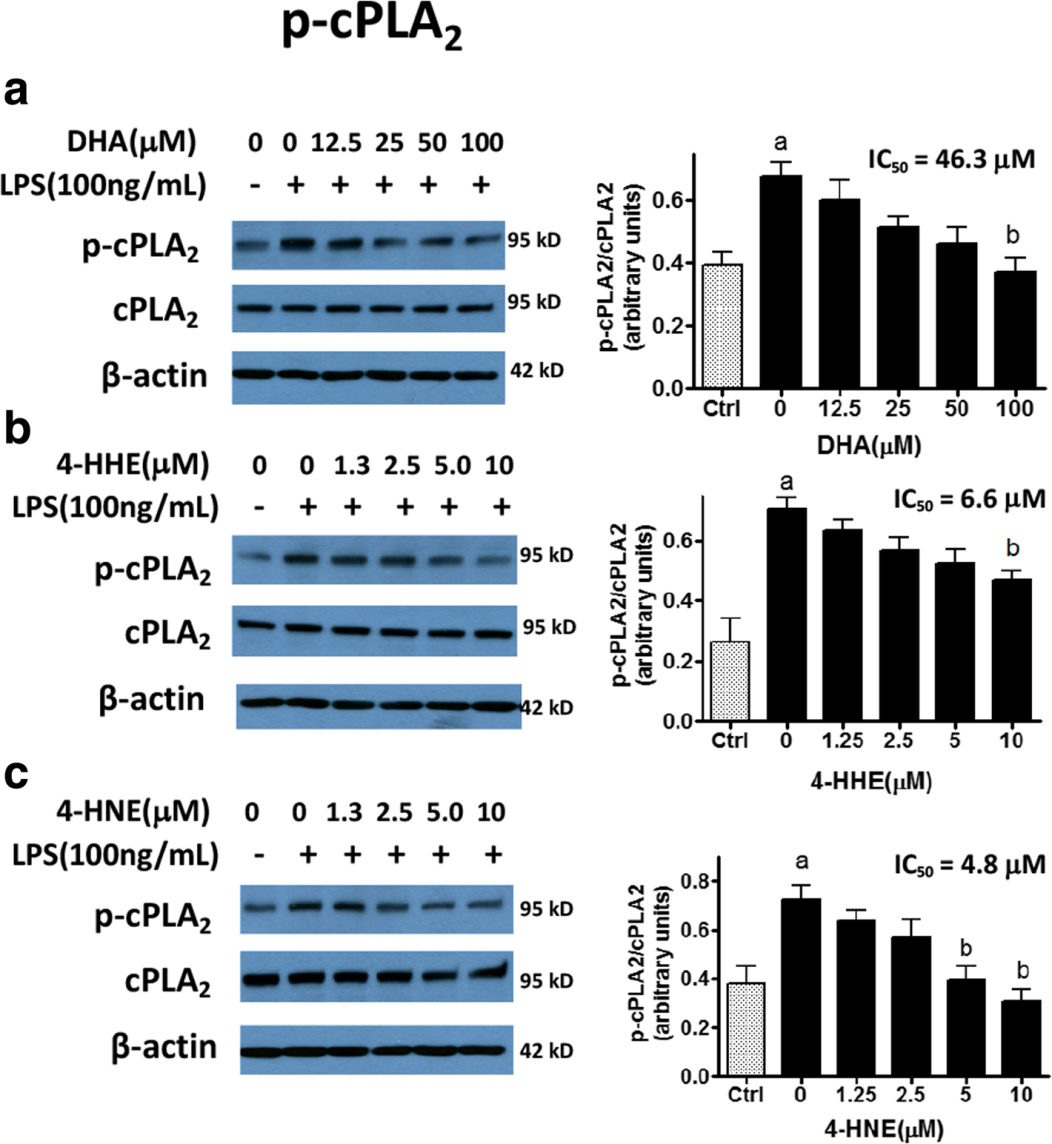
**a**–**c** Effects of DHA, 4-HHE, and 4-HNE on LPS-induced p-cPLA_2_ expression in microglial cells. Microglial cells were cultured in 12-well plates and pre-treated with DHA (12.5–100 μM), 4-HHE (1.25–10 μM), and 4-HNE (1.25–10 μM) for 1 h prior to stimulation with LPS (100 ng/mL) for 4 h. Protein extracts were used for Western blot analysis of p-cPLA_2_, total cPLA_2_, and β-actin as described in text. A representative blot was shown in each set of experiment. Bar graphs represent p-cPLA_2_/cPLA_2_ ratios from four experiments with different passages. Results are expressed as the mean ± SEM (*n* = 4) and analysis by one-way ANOVA followed by Bonferroni post-tests, “a” represents significant differences (*p* < 0.05) between control versus LPS, and “b” represents significant differences (*p* < 0.05) comparing test compounds with LPS. IC_50_ values for each test compound were determined using the formula for regression analysis in Microsoft Excel 2016

Results from earlier studies have demonstrated the ability of LPS to activate p-cPLA_2_ in microglial cells through the p-ERK1/2-dependent pathway [10, 29]. In this study, we tested the ability of DHA, 4-HHE, and 4-HNE to suppress LPS-induced p-cPLA_2_. Figure 4a–c shows that DHA (12.5–100 μM) as well as 4-HHE (1.25–10 μM) and 4-HNE (1.25–10 μM) suppressed LPS-induced p-cPLA_2_ expression in a dose-dependent manner. The IC_50_ values for DHA, 4-HHE, and 4-HNE are 46.3, 6.6, and 4.8 μM, respectively.

### DHA, 4-HHE, and 4-HNE upregulate the anti-oxidant response involving Nrf2 and HO-1

**Fig. 5:**
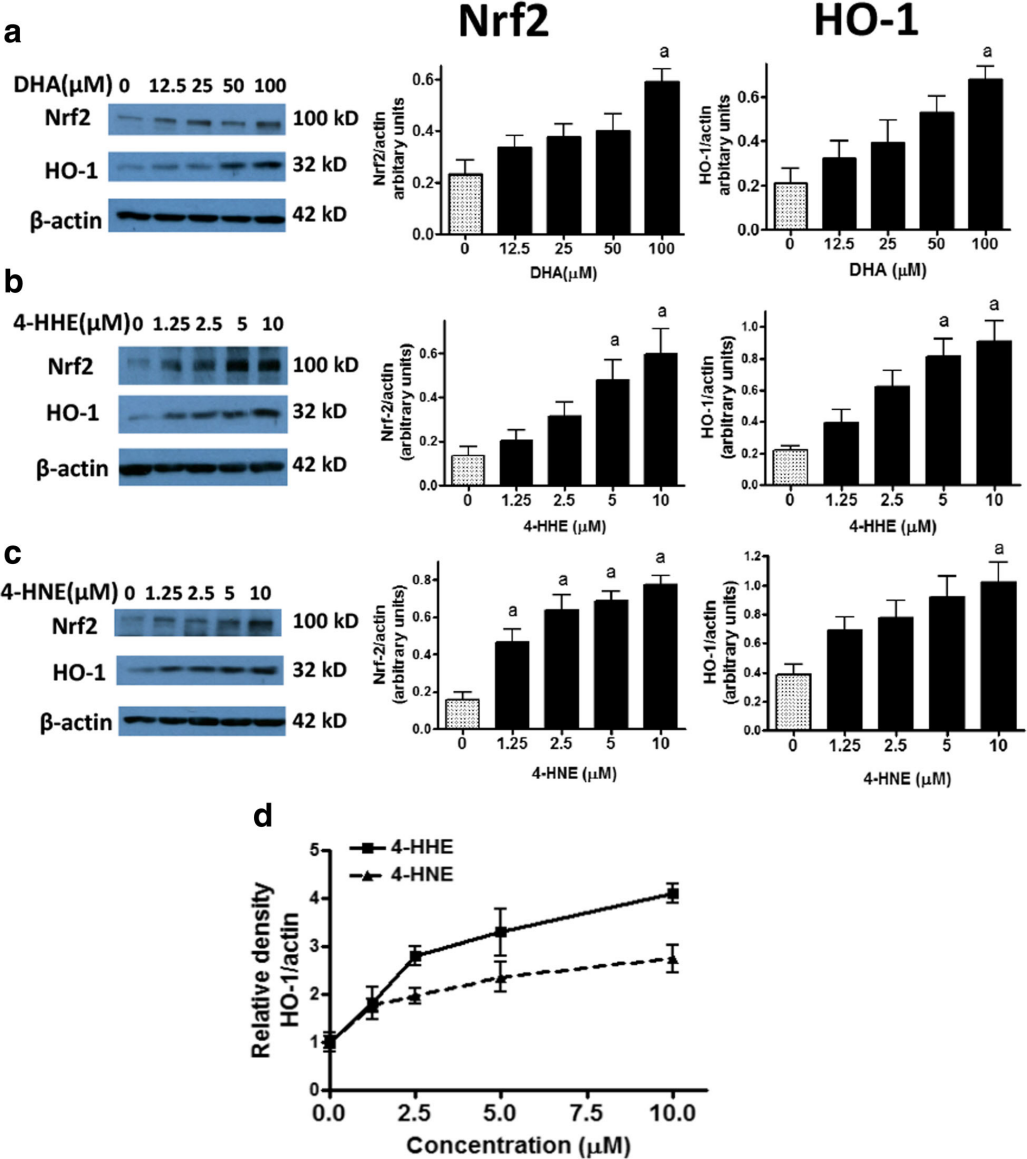
Effects of DHA, 4-HHE, and 4-HNE on the induction of Nrf2 and HO-1 protein expression in BV-2 microglial cells. Cells were cultured in 12-well plate dish and pre-treated with **a** DHA (12.5–100 μM), **b** 4-HHE (1.25–10 μM), and **c** 4-HNE (1.25–10 μM) for 1 h followed by stimulation with LPS (100 ng/mL) for 6 h. Representative Western blots are shown for effects of DHA, 4-HHE, and 4-HNE on Nrf2 and HO-1 expression with β-actin as loading control. Results are from four experiments with different passages. Bar graphs represent relative densities of Nrf2/β-actin or HO-1/β-actin ratios. Analysis by one-way ANOVA followed by Bonferroni post-tests; “a” represents significant differences (*p* < 0.05) comparing between control versus test compounds. **d** Data depict the fold increase in HO-1 protein expression comparing 4-HHE to 4-HNE

In our earlier studies, we observed that some botanical polyphenols, such as quercetin, not only suppressed LPS-stimulated NO and ROS production, but could also enhance the anti-oxidant pathway involving Nrf2 and production of the potent anti-oxidative enzyme HO-1 [46]. In the present study, DHA (12.5–100 μM) as well as 4-HHE (1.25–10 μM) and 4-HNE (1.25–10 μM) showed a dose-dependent increase in the expression of Nrf2 and HO-1 in microglial cells (Fig. 5a–c). A comparison of the effects of 4-HHE and 4-HNE on HO-1 expression showed that 4-HHE was more potent than 4-HNE in producing HO-1 (Fig. 5d).

### DHA and LPS regulate 4-HHE and 4-HNE levels in microglial cells

Studies so far have indicated anti-inflammatory and anti-oxidative effects of 4-HHE and 4-HNE added exogenously to microglial cells. Since these two alkenals are derived from two different PUFA with opposing functions, it seems important to examine whether these compounds are produced within the cells and how their production is regulated by LPS and DHA. First, we developed and validated the LC-MS/MS analysis of 4-HHE and 4-HNE. A summary of the validation is shown in Tables 1 and 2. The LC-MS/MS analysis was then used to simultaneous measure levels of 4-HHE and 4-HNE in microglial cells (10^6^ cells plated in 60 mm dish) which were pre-treated with DHA (50 μM) for 1 h and followed by stimulation with LPS (100 ng/mL) for 6 h. Initially, analysis of the culture medium after 6-h incubation indicated no alkenals were detected in the culture medium. Subsequently, after removing the culture medium, cells were harvested from the culture dish by scraping with 0.5 mL of PBS/methanol (1:1, *v*/*v*). The cell suspension was then transferred into an Eppendorf tube and was used for both protein determination and for LC-MS/MS analysis.

**Table 1:** Method validation parameters for detection of 4-HHE in microglia cells

Linear range (ng/mL)	Linearity	Matrix effect	Phree recovery	Sep-Pak recovery	Intraday		Interday	
					Accuracy (bias, %)	Precision (CV, %)	Accuracy (bias, %)	Precision (CV, %)
15.63–1000	0.9994	92.6%	104.4%	92.4%	0.5%	0.7%	1.5%	0.8%

*CV* coefficient of variance

**Table 2:** Method validation parameters for detection of 4-HNE in microglia cells

Linear range (ng/mL)	Linearity	Matrix effect	Phree recovery	Sep-Pak recovery	Intraday		Interday	
					Accuracy (bias, %)	Precision (CV, %)	Accuracy (bias, %)	Precision (CV, %)
6.25–250	0.9989	92.6%	88.8%	92.1%	2.4%	1.0%	9.9%	1.3%

*CV* coefficient of variance

### Effects of DHA and/or LPS on 4-HHE and 4-HNE in BV-2 microglial cells

**Fig. 6:**
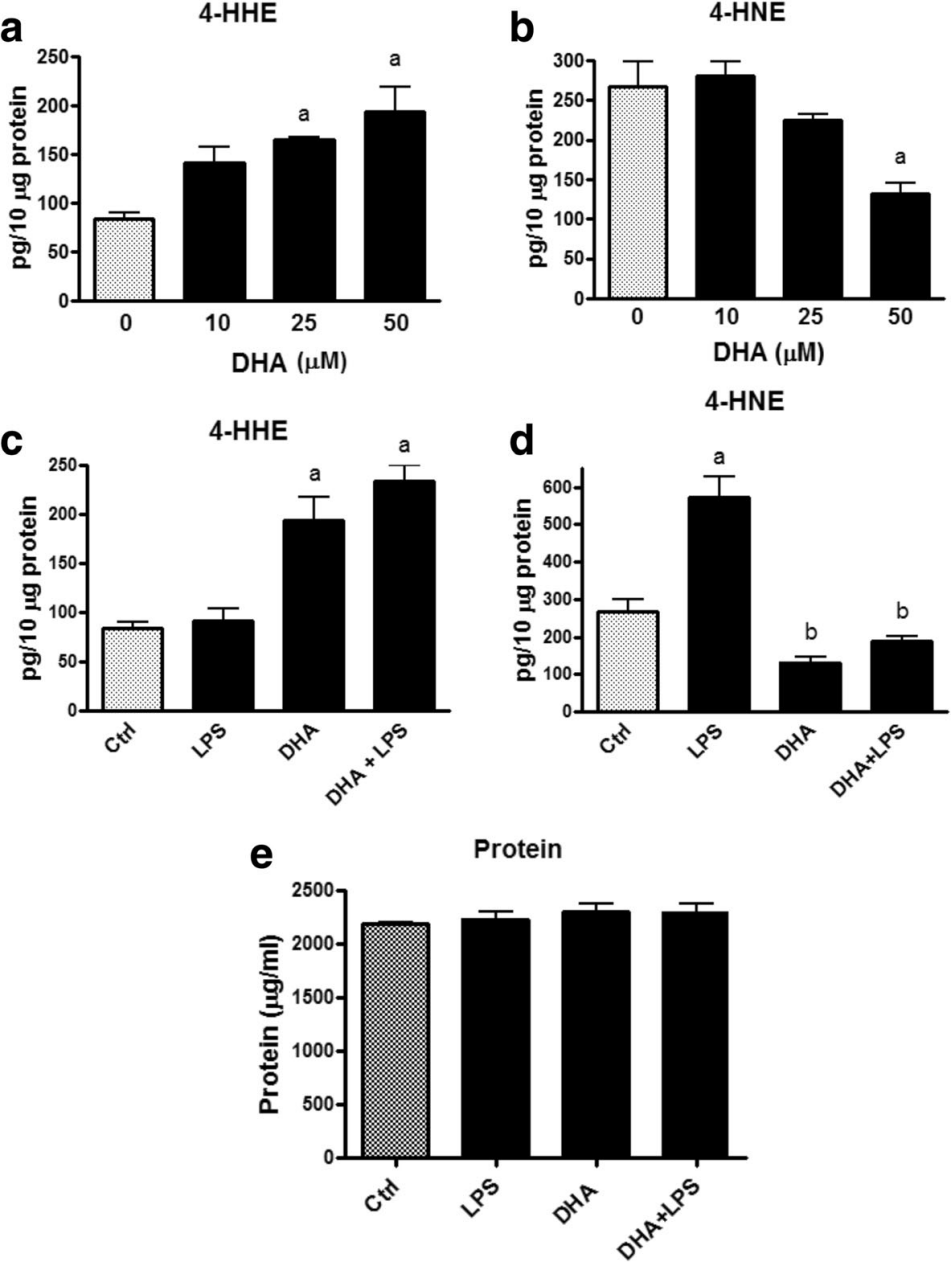
Effects of DHA and/or LPS treatment on 4-HHE and 4-HNE levels in microglia cells. **a**, **b** Cells were cultured in 60-mm dish and serum-starved for 3 h before addition of DHA (10, 25, and 50 μM) for 1 h and LPS (100 ng/mL) for 6 h. After treatment, culture medium was removed and cells were suspended with 0.5 mL of PBS:H_2_O (1:1, *v*/*v*). Cell suspension was vortexed and aliquots taken for LC-MS/MS measurement as well as protein assay as described in the “Methods” section. Results depict levels of 4-HHE (**a**) and 4-HNE (**b**) upon treatment with DHA and expressed as picogram per 10 μg of protein. Each value represents the mean ± SEM of three biological replicates with duplicate analysis. Analyzed by one-way ANOVA followed by Bonferroni post-tests; “a” represents significant differences (*p* < 0.05) comparing DHA groups with control group. Levels of 4-HHE (**c**) and 4-HNE (**d**) upon treatment of cells with LPS (100 ng/mL) and/or DHA (50 μM). One-way ANOVA followed by Bonferroni post-tests; “a” significant differences (*p* < 0.05) comparing test group with control group, and “b” represents significant differences (*p* < 0.05) comparing LPS group with DHA and DHA + LPS groups. **e** Protein concentration from four passages of microglia cells indicated no significant changes due to the different treatment conditions

Using the LC-MS/MS method, we first tested the effects of different doses of DHA (10–50 μM) on the levels of 4-HHE and 4-HNE in BV-2 microglial cells in the presence and absence of LPS (100 ng/mL). As shown in Fig. 6a, when cells were treated with DHA (10, 25, and 50 μM) for 7 h, there was a dose-dependent increase in levels of 4-HHE with significant increases (*p* < 0.05) at 25 μM or higher. Under these conditions, treatment with DHA resulted in a dose-dependent decrease in levels of 4-HNE with significant decrease (*p* < 0.05) at 50 μM (Fig. 6b). We further determined levels of 4-HHE and 4-HNE in cells treated with DHA (50 μM) and/or LPS (100 ng/mL). Figure 6c showed that when LPS is added after DHA, there is a small but no significant further increase in 4-HHE as compared with treatment with DHA alone (Fig. 6c). Cells stimulated with LPS showed a significant increase (*p* < 0.05) in levels of 4-HNE (Fig. 6d), albeit no change in the levels of 4-HHE (Fig. 6c). Furthermore, when cells were pre-treated with DHA and followed with LPS, the ability of LPS to increase 4-HNE was reduced (Fig. 6d). In this experiment, levels of 4-HHE and 4-HNE were expressed based on the amount of proteins in the cell samples and there was no observable change in protein levels for all the treatment conditions (Fig. 6e).

### Regulation of 4-HHE and 4-HNE by cPLA_2_ and iPLA_2_ inhibitors

**Fig. 7:**
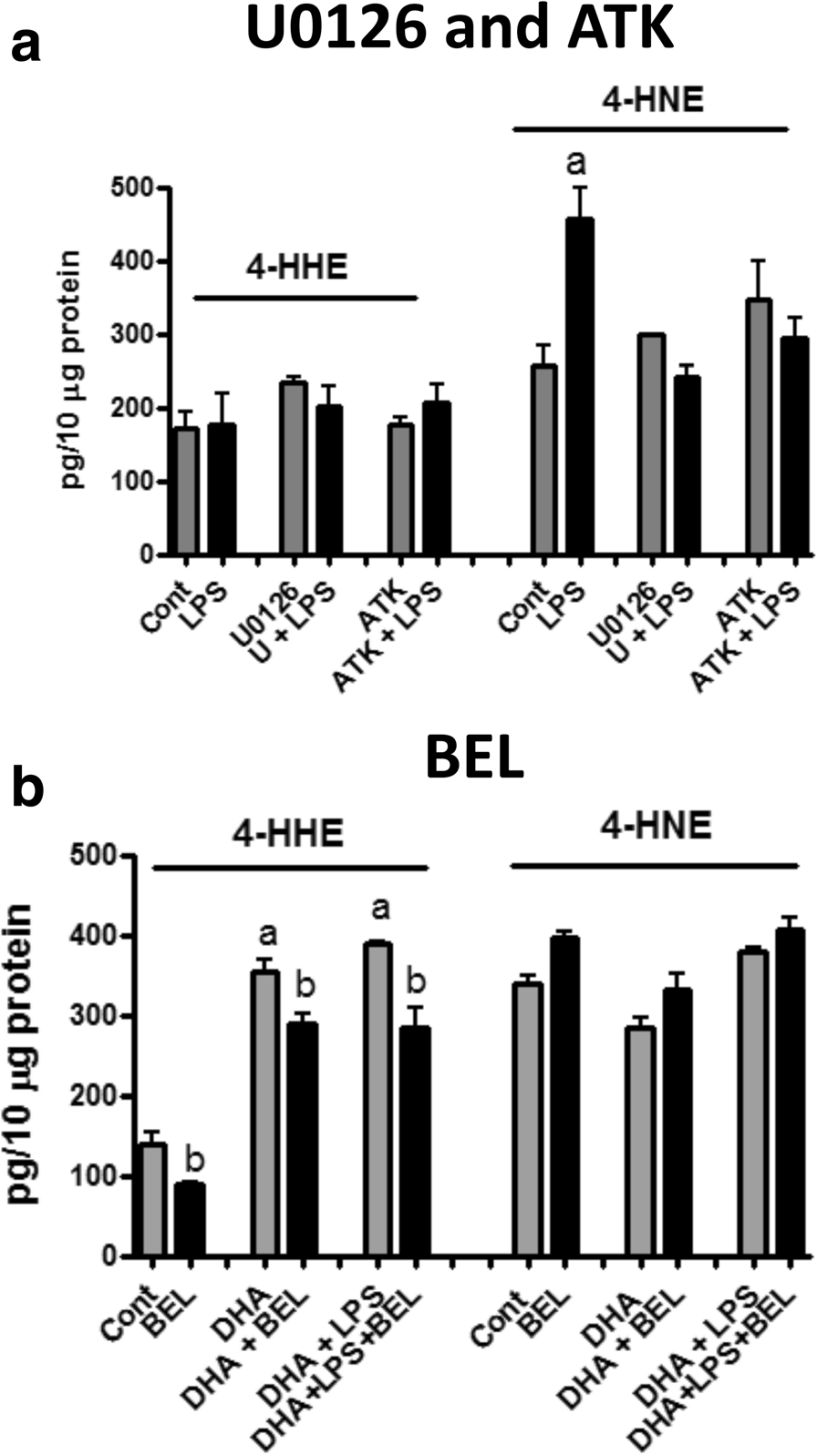
Effects of cPLA_2_ and iPLA_2_ inhibitors on levels of 4-HHE and 4-HNE in microglia cells treated with DHA and/or LPS. **a** Effects of cPLA_2_ inhibitors. Cells were pre-treated with U0126 (5 μM) or ATK (5 μM) for 1 h and followed by LPS (100 ng/mL) for 6 h. 4-HHE and 4-HNE levels were measured using the LC-MS/MS analysis as described in the “Methods” section. Each value represents the mean ± SEM of three biological replicates with duplicate analysis and is normalized to per 10 μg of protein. **b** Effects of iPLA_2_ inhibitor. Cells were pre-treated with BEL (5 μM) and /or DHA (50 μM) for 1 h prior to LPS (100 ng/mL) for 6 h. Results are representation of data from one cell passage; experiments repeated with different passages showed similar profiles. Analysis using one-way ANOVA followed with Bonferroni post-tests. In **a**, “a” represents significant increase (*p* < 0.001) in 4-HNE due to LPS stimulation as compared with control. In **b**, “a” represents significant increase (*p* < 0.01) in 4-HHE due to DHA and DHA + LPS compared with control and “b” represents significant decrease (*p* < 0.001) in 4-HHE due to BEL

Since our earlier studies provided evidence for LPS to stimulate p-ERK1/2 and subsequently phosphorylation of cPLA_2_ in primary and BV-2 cells, we surmise that the LPS-induced increase in 4-HNE is associated with activation of the LPS/p-ERK1/2/p-cPLA_2_ pathway, and in turn, the increase in ARA is subsequently subject to peroxidation to produce 4-HNE [29]. In order to test this hypothesis, cells were pre-treated with U0126 (5 μM), an inhibitor for the MEK1/2 and ERK1/2 pathway, and ATK (AACOCF3) (5 μM), an inhibitor of cPLA_2_, and followed by stimulation with LPS. As shown in Fig. 7a, both U0126 and ATK abrogated the LPS-induced increases in the 4-HNE levels, whereas the inhibitors alone did not alter basal levels of 4-HNE or 4-HHE.

Since the iPLA_2_ has been regarded as the responsible enzyme for the release of DHA from membrane phospholipids, we further investigated whether the increase in 4-HHE due to addition of DHA involves iPLA_2_. As shown in Fig. 7b, addition of BEL (5 μM), a specific inhibitor for iPLA_2_, significantly (*p* < 0.001) decreased the basal levels of 4-HHE in the cells, suggesting the involvement of iPLA_2_ in the metabolism of the endogenous DHA pool. On the other hand, although exogenous DHA resulted in increased levels of 4-HHE, this increase was only partially inhibited by BEL (Fig. 7b). Similarly, treatment of cells with DHA and LPS resulted in a small increase in 4-HHE as compared to treatment with DHA alone, the increase in 4-HHE was only partially inhibited by BEL (Fig. 7b). With a small decrease in 4-HNE due to the presence of DHA, BEL did not produce obvious changes in 4-HNE levels (Fig. 7b).

## Discussion

**Fig. 8:**
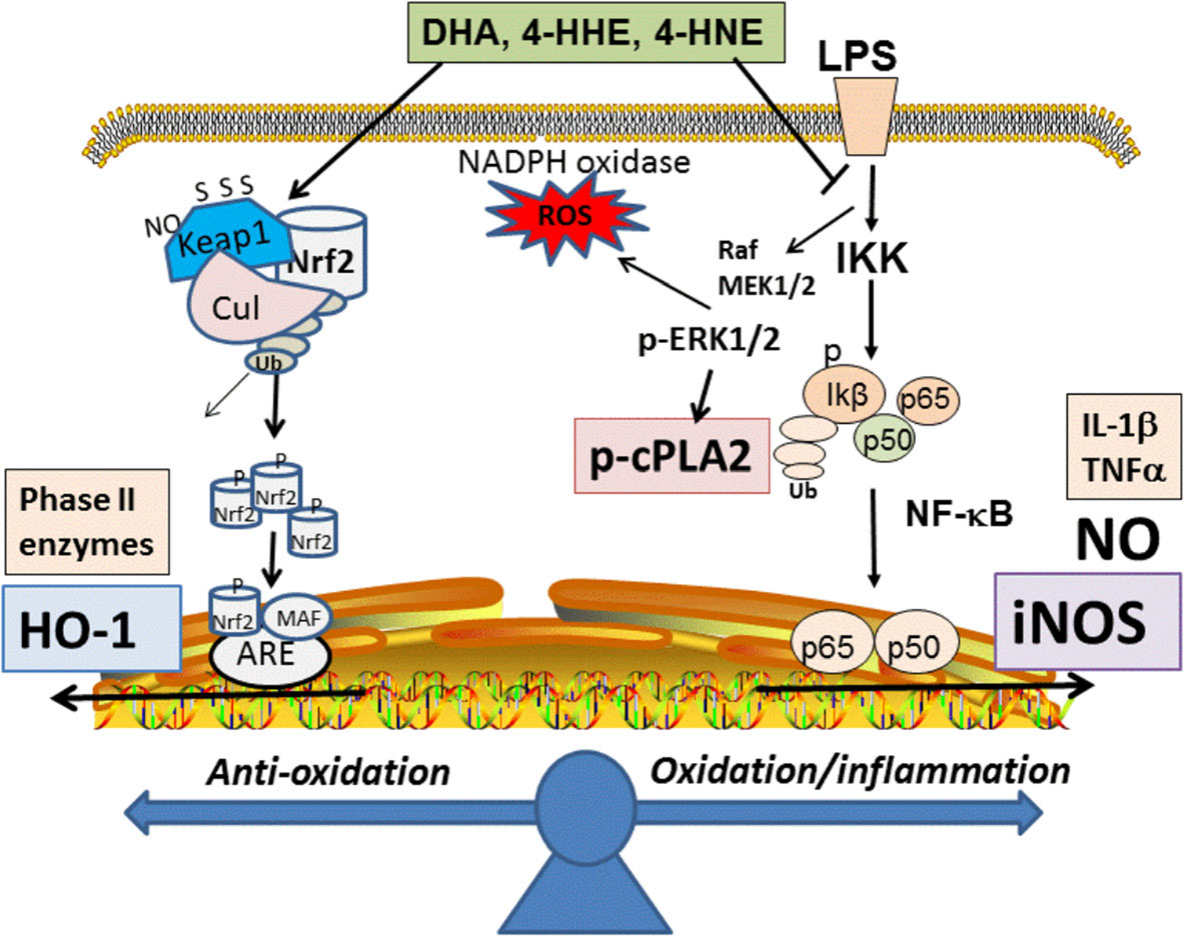
A scheme depicting effects of electrophiles on upregulation of the Nrf2/HO-1 pathway and downregulation of NO/ROS/cPLA_2_ pathways in microglial cells

In this study, we provided evidence that depending on concentration, DHA (12.5–100 μM) as well as 4-HNE (1.25–10 μM) and 4-HHE (1.25–10 μM) can suppress LPS-induced increase in production of NO, ROS, and p-cPLA_2_ in the BV-2 microglial cells. Also, with the same concentration ranges, these compounds can stimulate the Nrf2 anti-oxidant pathway and increased synthesis of HO-1. A scheme in Fig. 8 depicts the signaling pathways for these compounds acting as electrophiles to upregulate the Nrf2/HO-1 pathway and downregulate pathways for LPS-induced NO/ROS/cPLA_2_. Based on the IC_50_, the effects of 4-HNE and 4-HHE are five- to tenfold more potent than DHA. In this study, although both 4-HHE and 4-HNE showed similar effects on suppressing the NO/ROS/cPLA_2_ and upregulating the Nrf2 pathways, subtle differences can be observed. For example, based on IC_50_ values, 4-HNE is more potent than 4-HHE in suppressing LPS-induced p-cPLA_2_ (Fig. 4b). In addition, 4-HHE is more potent than 4-HNE in stimulation of HO-1 (Fig. 5d). This latter observation is in line with a report showing higher electrophilic activity for 4-HHE as compared to 4-HNE [6]. Taken together, results from our study with BV-2 microglial cells, as well as studies with smooth muscle and endothelial cells, clearly demonstrate that exogenous 4-HHE and 4-HNE are capable of enhancing the Nrf2 anti-oxidative activity and suppressing the LPS-induced oxidative/inflammatory responses [37–39]. The effective concentration ranges for these alkenals to enhance Nrf2/HO-1 and to suppress the LPS pathways are comparable to those observed with quercetin, a botanical polyphenol enriched in berries [31, 46]. In fact, due to the electrophilic properties of these alkenals, their ability to offer adaptive responses can be shown in a number of cell systems [28, 47]. Studies have also shown the ability of these alkenals to alter cell metabolism through forming adducts with proteins, nucleic acids, and phospholipids [16]. Obviously, more studies are needed to determine the physiological function of these alkenals generated within the cells.

Our ability to develop a sensitive LC-MS/MS method for simultaneous determination of 4-HHE and 4-HNE levels has produced interesting results regarding the underlying mechanism(s) for the regulation of these alkenals in microglial cells. In our protocol, the sensitivity of detection was improved by utilizing a SPE strategy to remove phospholipids which are a major source of matrix effects. When compared with traditional antibody-based methods, the analysis of free aldehydes avoids epitopes that are bound to target proteins or nucleic acids [48].

As shown in Fig. 6a, b, the LC-MS/MS methodology showed higher basal levels of 4-HNE as compared to 4-HHE (around 250 pg to 75 pg/10 μg protein, respectively, in 10^6^ cells cultured in the 60-mm dish). The higher endogenous levels of 4-HNE as compared with 4-HHE in these cells is consistent with results of the fatty acid analysis showing higher levels of ARA than DHA in these cells [34]. In the present study, stimulation of cells with LPS led to the increase in 4-HNE levels without altering the 4-HHE levels (Fig. 6c, d). The ability for LPS to induce an increase in 4-HNE is in agreement with previous studies linking LPS to p-ERK1/2 and p-cPLA_2_, and in turn, releasing ARA for lipid peroxidation [29]. Consequently, U0126 (an inhibitor for MEK1/2 and p-ERK1/2) and ATK (an inhibitor for cPLA_2_) were shown to abrogate LPS-induced 4-HNE (Fig. 7a). Based on these results, it can be assumed that measurement of 4-HNE can be used as a biomarker for oxidative and inflammatory responses in microglial cells.

**Fig. 9:**
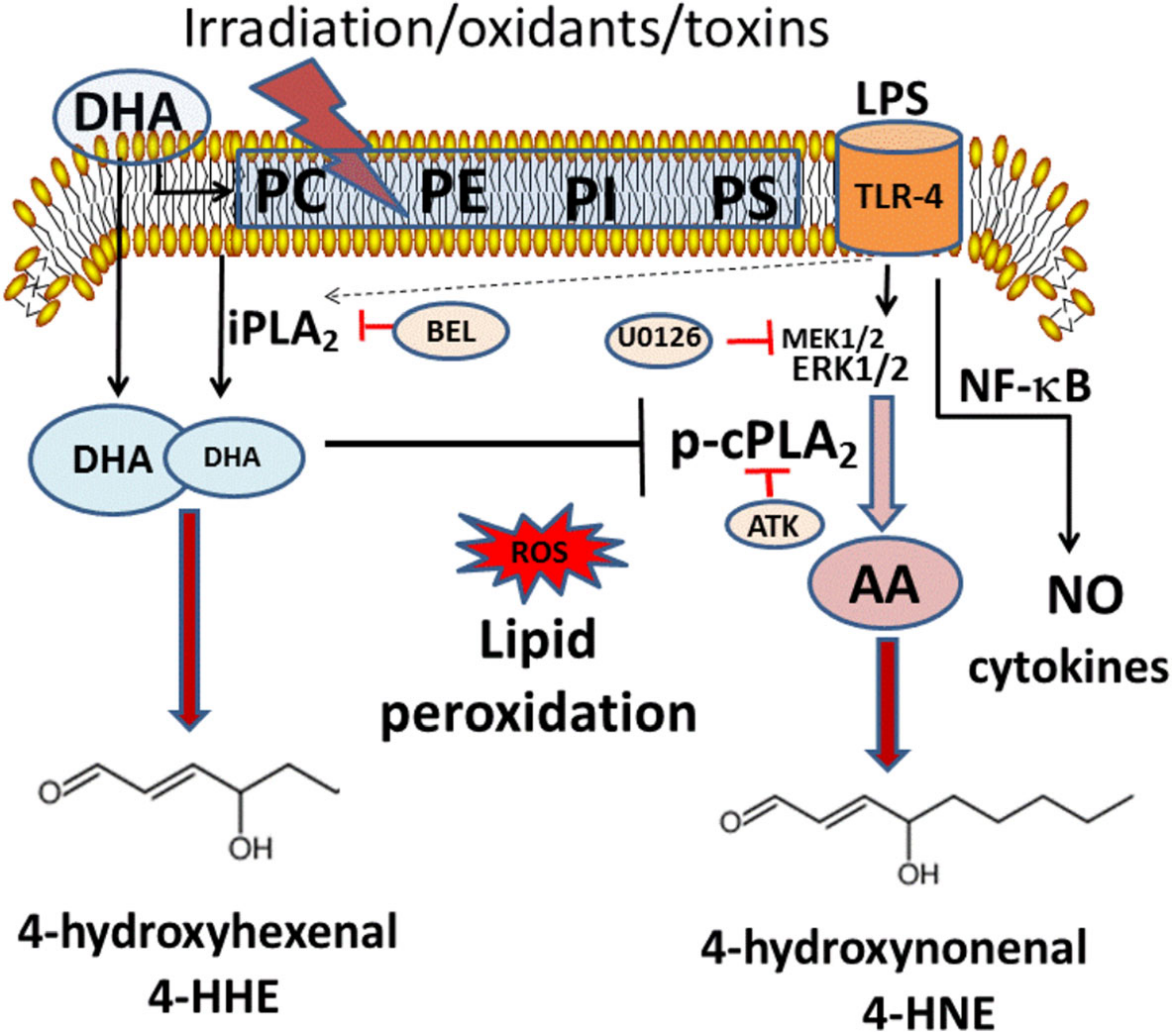
A scheme depicting metabolic pathways for production of 4-HHE and 4-HNE through different enzymatic (cPLA_2_ and iPLA_2_) and non-enzymatic (lipid peroxidation) mechanisms

In this study, supplementation of cells with DHA was shown to result in a dose-dependent increase in 4-HHE levels whereas this condition resulted in a concomitant decrease in the levels of 4-HNE (Fig. 6a, b). The mechanism for exogenous DHA to inhibit 4-HNE is not well understood but a possible explanation could be due to inhibition of cPLA_2_ which is responsible for the release of ARA and hence 4-HNE. This phenomenon can also explain the ability for DHA to inhibit the increase in 4-HNE induced by LPS (Fig. 6d). In this study, cells pre-treated with DHA and followed by stimulation with LPS did not greatly alter the levels of 4-HHE (as compared with those with DHA alone) (Fig. 6c). These results are in agreement with the observation that LPS caused the increases in 4-HNE through activation of cPLA_2_ and ARA and that this pathway is not related to the DHA-induced 4-HHE pathway (Fig. 9).

Since BEL is a specific inhibitor for iPLA_2_, its ability to decrease the basal levels of 4-HHE suggests that under normal physiological conditions, iPLA_2_ is active in providing the pool of DHA from membrane phospholipids. However, it is surprising that BEL only showed a small ability to suppress the increase in 4-HHE due to exogenous DHA (Fig. 7b). These results suggest that during this period of exposure (7 h), a large portion of exogenous DHA probably enters the cell without incorporation into the membrane phospholipids, and this pool is directly available for peroxidation (Fig. 9). This form of entry is in agreement with the study by Tremblay et al. who observed the ability of exogenous DHA to form intracellular lipid bodies which subsequently showed interplay with mitochondrial and other intracellular organelles [49]. A study by Ishikado’s group also showed a fourfold increase in 4-HHE levels upon incubating endothelial cells with DHA (75 μM) for 6 h [38]. Taken together, these results show that lipid peroxidation is an on-going process in the microglial cells and production of 4-HNE and 4-HHE is regulated based on the availability of ARA and DHA within the cells.

Similar to results of our studies, Lu et al. demonstrated ability for DHA to suppress IFNγ-induced inflammatory responses (TNFα, IL-6, NO, and COX-2), as well as increases in HO-1 levels in BV-2 microglial cells [36]. However, relatively high levels for DHA are required to exert these effects. It remains to be further tested whether under these conditions, DHA acts as an electrophile to interact with components of the Nrf2 and NF-κB pathways. It is also possible that DHA, being a highly unsaturated fatty acid, may alter cell surface receptors and thus their downstream pathways [35]. In our earlier study, ARA and DHA were shown to alter membrane properties and activity of amyloid precursor protein in neuronal cells [50]. There is also evolving evidence showing protective effects of DHA due to the hormetic and stress response of 4-HHE in stimulating the Nrf2/HO-1 pathway [20, 27, 38, 51, 52]. However, further studies are needed to test whether this phenomenon can be demonstrated in the brain and other organs.

## Conclusions

In summary, our study indicated effects of DHA, 4-HHE, and 4-HNE to enhance Nrf2/HO-1 and mitigate LPS-induced NO, ROS, and cPLA_2_ responses in BV-2 microglial cells. LC-MS/MS analysis indicated measurable endogenous levels of 4-HNE and 4-HHE in microglial cells, and treatment with DHA resulted in the increases in 4-HHE levels, whereas, treatment with LPS resulted in the increases in 4-HNE levels through a cPLA_2_-dependent pathway. These measurements demonstrate that lipid peroxidation can be mediated through intracellular redox activity. These results provide a cell model to investigate how lipid peroxidation products may be regulated through intracellular signaling pathways associated with neuroinflammatory responses [53].

## Abbreviations

4-HHE: 4-Hydroxyhexenal
4-HHE-d_3_: 4-Hydroxyhexenal-d_3_
4-HNE: 4-Hydroxynonenal
ARA: Arachidonic acid
ATK: Arachidonyl trifluoromethyl ketone
BCA: Bicinchoninic acid
BEL: Bromoenol lactone
COX-2: Cyclooxygenase-2
cPLA_2_: Cytosolic phospholipase A_2_
DHA: Docosahexaenoic acid
DMEM: Dulbecco’s modified Eagle’s medium
DMSO: Dimethyl sulfoxide
FBS: Fetal bovine albumin
HO-1: Heme oxygenase-1
IFNγ: Interferon-γ
iNOS: Inducible nitric oxide synthase
iPLA_2_: Ca^2+^-independent phospholipase A_2_
LC-MS/MS: Liquid chromatography-tandem mass spectrometry
LLOQ: Lower limit of quantification
LOX: Lipoxygenase
LPS: Lipopolysaccharide
MAPKs: Mitogen-activated protein kinases
MRM: Multiple reaction monitoring
NF-κB: Nuclear factor kappa-light-chain-enhancer of activated B cells
NO: Nitric oxide
Nrf2: Nuclear factor E2-related factor 2
PBS: Phosphate-buffered saline
PLA_2_: Phospholipases A_2_
PUFAs: Polyunsaturated fatty acids
ROS: Reactive oxygen species
SD: Standard deviation
SDS-PAGE: Sodium dodecyl sulfate-polyacrylamide gel electrophoresis
SEM: Standard error of mean
SPE: Solid-phase extraction
TBS: Tris-buffered saline
